# Utility and challenges of using whole‐genome resequencing to detect emerging insect and mite resistance in agroecosystems

**DOI:** 10.1111/eva.13484

**Published:** 2022-10-09

**Authors:** Megan L. Fritz

**Affiliations:** ^1^ Department of Entomology University of Maryland College Park Maryland USA

**Keywords:** genome scanning, insects, mites, pesticide resistance evolution, resistance monitoring, temporal genomic change

## Abstract

Arthropods that invade agricultural ecosystems systematically evolve resistance to the control measures used against them, and this remains a significant and ongoing challenge for sustainable food production systems. Early detection of resistance evolution could prompt remedial action to slow the spread of resistance alleles in the landscape. Historical approaches used to detect emerging resistance included phenotypic monitoring of agricultural pest populations, as well as monitoring of allele frequency changes at one or a few candidate pesticide resistance genes. In this article, I discuss the successes and limitations of these traditional monitoring approaches and then consider whether whole‐genome scanning could be applied to samples collected from agroecosystems over time for resistance monitoring. I examine the qualities of agroecosystems that could impact application of this approach to pesticide resistance monitoring and describe a recent retrospective analysis where genome scanning successfully detected an oligogenic response to selection by pesticides years prior to pest management failure. I conclude by considering areas of further study that will shed light on the feasibility of applying whole‐genome scanning for resistance risk monitoring in agricultural pest species.

## 1 INTRODUCTION

Since the advent of modern agriculture, humans have competed with other species for food and fiber. To eliminate this competition, myriad physical, chemical, and biological control measures have been applied in agricultural ecosystems (agroecosystems). Melander (1914) was the first to recognize that when these competition‐reducing measures, collectively called “pest management,” are applied repeatedly, some pest populations evolve resistance to their effects. Although the pest management tools used in agroecosystems have changed since Melander's original work, the propensity of pests to evolve resistance has not. Today, resistance is defined as a genetically‐based decrease in the susceptibility of a population to a pest management tool that results from exposure in the field (Tabashnik et al., 2013; Walsh et al., 2022).

**FIGURE 1:**
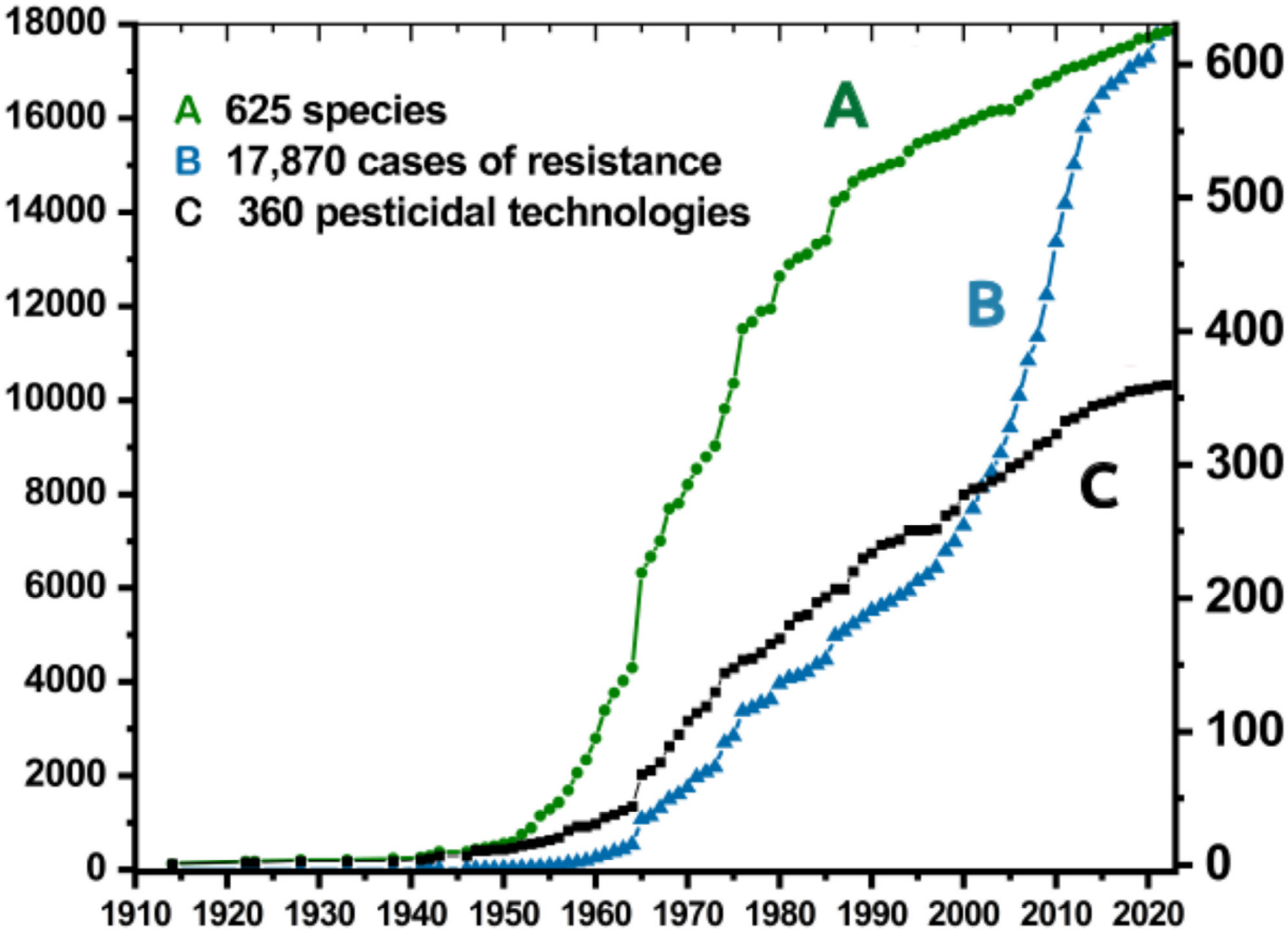
Evolution of arthropod resistance to xenobiotics from 1914 to 2022: Species, pesticidal technologies, and cases of resistance. Data courtesy of David Mota‐Sanchez and John C. wise. The arthropod pesticide resistance database. Michigan State University. Accessed April 18, 2022, at www.pesticideresistance.org.

Pesticide resistance was recently described as a “wicked problem,” partly because its complex underlying biological, sociological, and economic drivers make it difficult to prevent (Gould et al., 2018). Throughout this review, I use the terms “pesticide” and “pesticidal” to refer to any management approach that decreases arthropod survivorship and reproduction. To date, at least 625 arthropod species have evolved resistance to 360 pesticidal technologies (Figure 1). Repeated pesticide resistance emergence is challenging for farmers who bear increased crop management costs and for scientists working to replace ineffective technologies. In some arthropod species, unmanageable levels of pesticide resistance have resulted in abandonment of control programs and even cropping systems (Mallet, 1989; Wilson et al., 2018).

Recognition of these challenges has led the agricultural research community and governmental regulatory agencies to develop and deploy resistance management plans to improve pesticide durability (US Environmental Protection Agency [EPA], 1998; Wilson et al., 2018). Such plans include (1) pesticide resistance prevention measures deployed at the time of commercial release for novel pesticides (Box 1), (2) monitoring approaches used to identify pest resistance, and (3) remediation strategies to slow or reverse the spread of pest resistance, once detected (Box 2). A relevant example is the 1998 US EPA's mandate of a resistance management plan for transgenic crops that express insect‐specific toxins derived from *Bacillus thuringiensis* (Bt). This plan was adopted following the commercial release of Bt crops. It included resistance prevention measures like the production of Bt‐expressing cultivars that produced a “high dose” of toxin to kill >99% of target insects heterozygous for any resistance alleles (US EPA, 1998), and the planting of refugia, which promotes the survival of Bt‐susceptible individuals to mate with resistant conspecifics, thereby reducing the frequency of resistance alleles (Head & Greenplate, 2012; Roush, 1997). As an additional resistance prevention measure, multi‐toxin cultivars were made available when Bt toxins with novel modes of action were identified.

BOX 1 **Pesticide resistance prevention**

The importance of pesticide resistance prevention and management was recognized in the 1970s, as key pesticides used to manage a broad range of arthropod taxa began to lose efficacy (Brattsten et al., 1986; Koehler, 1974; Mallet, 1989; Sparks, 2013). Simultaneously, novel pesticide discovery costs soared (Sparks, 2013), underscoring the need to preserve remaining effective technologies and consider the durability of newly developed technologies. Today, multiple strategies for pesticide resistance prevention have been proposed and sometimes are used in combination. These approaches aim to relax selection pressure on individuals bearing resistance alleles and conserve susceptible alleles in arthropod populations.

**Establishment of refugia**

Refugia comprise host plants and often crops cultivated without protection by pesticidal technologies. The goal is to maintain susceptible alleles within a pest population. Impacts of the refuge strategy for resistance prevention are most potent when resistance is recessive, and individuals bearing resistance alleles incur a fitness cost in the absence of pesticide exposure (reviewed in Gould, 1998). Under these circumstances, refugia should maintain or even promote increases in susceptible allele frequencies as susceptible individuals emerging from an unprotected refuge will be numerous and have a reproductive fitness advantage over individuals bearing resistance alleles (Gould, 1998). While the EPA has mandated the planting of refugia to prevent resistance evolution to plant‐incorporated protectants (PIPs; US EPA, 1998), their use has also been proposed to prevent resistance to sprayable pesticidal compounds (Brattsten et al., 1986; Comins, 1977). In the case of PIPs, refugia can be planted in blocks adjacent to crops expressing PIPs (i.e., structured refuge) but are sometimes integrated into plantings of PIP expressing crops in the same field (i.e., a refuge in a bag; Yang et al., 2015).

**Dosage and timing of pesticide application**

The goal of pesticide application in some agricultural systems is to minimize economic losses, not prevent all pest damage. Under these circumstances, targeting a pest population for management is only necessary when it reaches a population size threshold resulting in economically damaging yield losses (Mumford & Norton, 1984). Fewer applications of pesticides are needed when these thresholds are considered, relaxing selection pressure on individuals bearing resistance alleles. Dosage per application must also be considered, as dosages that are too low may provide a fitness advantage to heterozygous individuals for resistance alleles (reviewed in Gould, 1998). Applying dosages above what is required to kill these heterozygotes reduces the frequency of resistance alleles in pest populations by eliminating heterozygous individuals. Interestingly, the high dose/refuge strategy mandated at the time of the first PIP commercial release (US EPA, 1998, 2001) combined both dosage considerations and the establishment of refugia, which has prevented PIP resistance in multiple major pest species for decades (Tabashnik & Carrière, 2017).

**Rotational use of pesticidal technologies**

Alternate applications of pesticidal technologies with different modes of action can provide a way to alleviate selection pressure on alleles that enhance resistance to both technologies (Georghiou et al., 1983). IRAC (2012) has supported this approach due to the practicality of its implementation.

**Combination of sprayable pesticidal technologies**

Simultaneous application of two pesticidal technologies according to manufacturer instructions relaxes selection pressure on individuals bearing resistance alleles by “redundant killing” (REX Consortium, 2013). When resistance alleles are at a low frequency, the probability that any individual bears alleles for resistance to two technologies is minimal. Under these circumstances, simultaneously applying two pesticides should kill most individuals in a pest population twice because each individual is susceptible to both pesticides. When an individual bearing resistance alleles survives the first pesticidal technology, the second technology provides a means for preventing the resistant individual from passing on their gametes. In rare cases, one pesticide may be combined with a synergist rather than a second pesticide, particularly if resistance is predicted to be metabolic (Brattsten et al., 1986).

BOX 2 **A case of successful resistance remediation**

Resistance remediation involves changes to agricultural practices that slow or reverse the spread of pesticide resistance. The best‐documented case of resistance remediation following EPA requirements occurred in 2006. Field failures of Cry1F‐producing maize (Herculex^®^ I containing event TC1507, Dow AgroSciences, and Pioneer Hi‐Bred International) were documented in regions of Puerto Rico, and follow‐up laboratory studies confirmed resistance ratios of >1000 in field‐collected *Spodoptera frugiperda* populations (Storer et al., 2010). Per EPA requirements, Dow AgroSciences withdrew the product from Puerto Rico and re‐examined resistance in *S. frugiperda* 4 years later. While Puerto Rican populations remained highly resistant to Cry1F after 4 years in the absence of Cry1F selection pressure, resistance did not spread to regions of the southeastern US where the subsequent use of crops that produced Cry1F was uncommon (Storer et al., 2012).

Many target insect species meeting the high dose resistance prevention criteria remain susceptible to Bt toxins, even 25 years after the first commercial release of Bt‐expressing crops, highlighting the success of carefully implemented resistance prevention approaches. Nevertheless, resistance has emerged in insect species that did not (Tabashnik & Carrière, 2017). While monitoring approaches and remediation strategies were also mandated as part of this plan (US EPA, 1998), available tools used to monitor pest populations sometimes provided ambiguous evidence of resistance and its potential to become an economic problem. It is unclear whether cases of resistant populations that arose could have been identified early and the spread of resistance prevented with improved monitoring.

Thus, as the evolution of pest resistance outpaces pesticide discovery (Sparks, 2013), it is important to consider the role that monitoring plays in sustaining the efficacy of existing and future pesticidal technologies. Ideally, monitoring should reveal changes in the resistance status of pest populations prior to a reduction in pesticide efficacy (i.e., “practical resistance”; Tabashnik et al., 2013). The current lack of these approaches is especially challenging because measuring the efficacy of both resistance prevention (Box 1) and remedial actions (Box 2) relies on the ability to detect changes in resistance status through monitoring.

Recently, there have been calls for studies to develop and test genomic monitoring approaches. The idea is that subtle responses to selection in pest genomes could be detected on short time scales before resistance reaches levels that cause widespread crop failure (Gould et al., 2018; US EPA, 2018). Modern “omics” tools should be sensitive enough to measure early changes in allele frequency resulting from pesticide exposure, thereby signaling growing resistance. Allele frequency changes could then be used to trigger remedial action aimed at slowing the spread of resistance. Here, I describe genomic approaches to uncover pesticide resistance mechanisms, strategies currently used for pesticide resistance monitoring, and novel genome‐scale approaches currently used to detect signatures of selection in natural systems. I conclude by considering the potential and pitfalls of extending genome‐scale approaches used in natural systems to a predictive framework for identifying emerging pesticide resistance and mitigating its spread.

## 2 MOLECULAR GENETIC MECHANISMS OF PESTICIDE RESISTANCE

Survival and reproduction of arthropod pests are limited by applying pesticide sprays, deployment of crops expressing plant‐incorporated protectants (e.g., Bt Crystalline [Cry] toxins and dsRNA), crop rotation, and mechanical soil disruption. This diversity of pest management practices results in diverse mechanisms by which pests evolve resistance. Although resistance mechanisms can be characterized according to biochemical and molecular genetic mechanisms, the latter provides a unifying framework for classifying resistance evolution in response to diverse management approaches (Feyereisen et al., 2015). According to the process of natural selection, any genetic mutation that reduces pesticidal impacts will be favored in arthropod populations and therefore change in frequency over time. Common classes of resistance‐conferring mutations include (1) nonsynonymous single nucleotide polymorphisms (SNPs) and insertion/deletion polymorphisms (indels) in genes encoding pesticide target sites, (2) SNPs and indels that impact gene regulation, and (3) indels that modify gene copy number. Nonsynonymous SNPs and indels modify the amino acid sequence and structure of proteins expressed from pesticide target‐site genes, impacting pesticide binding and resulting in loss of efficacy. A change in cis‐ or trans‐regulatory gene sequences may increase pesticide resistance if receptor gene expression is reduced or the expression of genes responsible for pesticide metabolism, excretion, and sequestration is increased. Likewise, indels that increase metabolic gene copy number can increase gene expression and pesticide metabolism when all gene copies remain functional.

Theory suggests that the number and effect size of these resistance‐causing mutations vary according to available genetic variation in a pest population, mutational constraints on pest genes, and the strength of selection imposed by pesticides (McKenzie, 2000). Monogenic resistance caused by a single and initially rare mutation of large effect, often in a target‐site gene, should be favored when the dosage of pesticide experienced by a pest requires a resistance phenotype outside of and many times greater than the initial distribution of resistance phenotypes in a naive pest population. Resistance may result from many mutations of small effect (i.e., polygenic resistance) if the pesticide dosage experienced by naive populations requires resistance phenotypes within their current phenotypic distribution (Ffrench‐Constant et al., 2004). Resistance‐causing mutations of both large and small effect sizes can accumulate in pest genomes, leading to heightened resistance to a pesticidal compound (Duneau et al., 2018; Tian et al., 2011; Taylor et al., 2021). Modeling predicts that large‐effect mutations will increase in frequency before those of smaller effect size if they are already present in pest populations before selection (Groeters & Tabashnik, 2000). However, in some cases, mutations of large effect arise after pesticide use is widespread. Under these circumstances, the initial phenotypic distribution of resistance in the pest population, pesticide dosage, as well as degree of dominance and fitness costs of resistance mutations should govern the order with which mutations of large and small effect size increase in frequency, where high doses favor large effect mutations to increase first (Groeters & Tabashnik, 2000; McKenzie & Batterham, 1994). This order effectively delays resistance and is the impetus for the “high dose” pesticide prevention measure integrated into Bt resistance management plans.

Decades of previous studies have revealed mutations in genes encoding pesticide target sites (Dermauw et al., 2012; Dong & Scott, 1994; Gahan, 2001; González‐Cabrera et al., 2013; Tao et al., 2013; Troczka et al., 2012), genes responsible for metabolic resistance (Daborn et al., 2007; Joußen et al., 2012; Rooker et al., 1996), and genes conferring behavioral (Knolhoff et al., 2006; Mabry & Spencer, 2003) and morphological changes (Wood et al., 2010). The resistance‐causing mutations for many pest species and specific pest management approaches have been extensively reviewed elsewhere (e.g., Feyereisen et al., 2015; Jurat‐Fuentes et al., 2021; Li et al., 2007; Scott, 2017; Van Leeuwen & Dermauw, 2016). Although I identify specific illustrative examples of selection for pesticide resistance, my review does not aim to describe the extent of knowledge about biochemical or molecular genetic mechanisms of pesticide resistance. Rather, my goal is to review the historical context and evidence for applying genomic approaches to resistance monitoring in wild pest populations.

## 3 GENOMIC APPROACHES FOR DETECTING PESTICIDE RESISTANCE MECHANISMS

Genetic and genomic tools have played a major role in discovery of pesticide resistance mechanisms. When resistance is detected in a pest population, quantitative genetic and genomic experiments can link changes in a pest's resistance status to one or more causal mutations across the genome (i.e., genome architecture). Commonly used approaches include quantitative trait locus (QTL) analysis (e.g., Gahan et al., 2001; Heckel et al., 1999; Taylor et al., 2021; Wondji et al., 2007), bulked segregant analysis (BSA; e.g., Benowitz et al., 2022; Fotoukkiaii et al., 2021; Van Leeuwen et al., 2012), and evolve and resequence studies (Snoeck et al., 2019; Wybouw et al., 2019). QTL and BSA approaches use offspring from planned crosses between pesticide‐resistant and susceptible populations and SNPs from across the genome to identify genomic regions where genotypic variation is strongly associated with variation in resistance phenotypes. Evolve and resequence approaches typically start with a genotypically diverse population and proceed with selection for higher levels of pesticide resistance in replicate populations over many generations. Pooled DNA from selected individuals is sequenced, and genotypes at SNPs across the genome are compared to those of unselected replicate populations to identify genomic regions showing the greatest allele frequency differences. While all three of these approaches have been instrumental in identifying genes associated with resistance, they are not stand‐alone tools for detecting emerging resistance. QTL and BSA approaches require the use of populations for which resistance has already been established. Furthermore, evolve and resequence studies rely on laboratory selection for increased pesticide resistance, which may not recover the same resistance mechanisms as those arising from field exposure (Ffrench‐Constant, 2013). Detecting emerging resistance in agroecosystems requires coupling allele frequency changes over generations to subtle declines in pest susceptibility.

## 4 ADVANTAGES OF EARLY DETECTION THROUGH MONITORING

Monitoring pest populations prior to and following the deployment of a novel pesticidal technology is critical to detecting heritable changes in susceptibility that require remedial actions to reduce survivorship and reproduction of resistant pests. While monitoring has been implemented to detect emerging resistance for a number of pests, documentation of successful resistance remediation resulting from monitoring is limited (Box 2). As one example, pest resistance to Bt toxins has been carefully examined, because the US EPA required remedial action plans for industry registrants of new Bt technologies. In theory, these plans involved informing customers and extension agents in affected areas, implementing alternative means to control resistant populations, increasing monitoring in affected areas, and ceasing sales in affected and bordering counties (US EPA, 1998, p. 66). The best‐documented case of resistance remediation following EPA requirements was in 2006, after *Spodoptera frugiperda* populations evolved high levels of resistance to Cry1F‐producing maize (Storer et al., 2010; Box 2). Successful remediation in this system demonstrated there is a resistance allele frequency above which it was difficult to restore local susceptibility through remedial action and highlighted the importance of early resistance detection. Nevertheless, when resistance was detected, reducing selection pressure at the site of resistance evolution helped slow its spread and extend the useful life of this management technology. Few other examples of successful remediation are available, partly due to disagreements over how to use data from current monitoring approaches to trigger remediation (US EPA, 1998, p. 40).

## 5 CURRENT PESTICIDE RESISTANCE MONITORING APPROACHES

Early work on the mechanisms of pesticide resistance emphasized single genetic loci as the primary targets of selection in agroecosystems (Ffrench‐Constant et al., 2004; McKenzie & Batterham, 1998). Genetic mutations encoding changes to amino acid sequences and protein structure were thought to be the dominant mechanism of resistance evolution. Under selection by pesticides, these mutations conferred a fitness advantage but were also thought to impose a fitness cost in the absence of pesticides. Thus, resistance mutations were thought to remain at very low frequencies until selection occurred by pesticidal application. This single gene paradigm resulted in monitoring efforts focused on detecting monogenic resistance, although some consideration was given to polygenic resistance (Via, 1986). Current monitoring approaches may quantify phenotypic variation in pesticide tolerance levels and/or the frequencies of resistance‐conferring mutations in field‐collected populations (Table 1).

**TABLE 1:** Monitoring approaches to detect growing pesticide resistance in pest populations

Method	Example publications	Description
Dose–response or discriminating dose bioassays	Ali et al. (2006), Bergh et al. (1999), Luttrell et al. (2004), Monnerat et al. (2015), Olson et al. (2000), Pietrantonio et al. (2007), Trouiller (1998), Vassiliou and Kitsis (2013)	Quantify differences in survivorship of field‐collected populations relative to susceptible populations
Feeding and dose‐development bioassays	Ali and Luttrell (2009), Cabrera et al. (2011), Dively et al. (2016), Luttrell and Jackson (2012)	Quantify differences in feeding and growth of field‐collected populations relative to susceptible populations
Crop yield or damage assessments	Dively et al. (2016, 2021), Reisig and Reay‐Jones (2015), Schmidt‐Jeffris et al. (2021), Yang et al. (2019)	Monitor levels of crop damage or reduced yields in the presence/absence of the pesticide
Molecular monitoring of candidate resistance genes	Benito‐Murcia et al. (2021), González‐Cabrera et al. (2013), Morin et al. (2004), Tabashnik et al. (2006), Gahan et al. (2007), Zhang et al. (2013), Jin et al. (2018)	Track changes in the frequency of known resistance‐conferring mutations following exposure to pesticide
F1/F2 screening	Andow and Alstad (1998), Génissel et al. (2003), Gould et al. (1997), Yang et al. (2018, 2020)	Quantify resistance in the F1 and F2 progeny of isofemale lines established from large field collections

### 5.1 Laboratory bioassays and damage assessments

Phenotypic differences among field‐collected pest populations have been documented with simple dose/mortality or more sensitive dose/development assays in laboratory settings (Heim et al., 1990; Luttrell et al., 2004; Tabashnik et al., 1987; Van Timmeren et al., 2019), often alongside a susceptible control population. If resistance is monogenic, recessive, and rare, as is often thought to be the case for target‐site mutations of major effect, documenting early changes to resistance status in field populations is challenging due to the relative scarcity of resistant individuals when novel pesticides are introduced. It is laborious to sample and bioassay sufficient pests for resistance detection, particularly when such rare, recessive target‐site mutations are conventionally thought to start at frequencies as low as 10^−6^ (Gould, 1998). If resistance is due to accumulation of many mutations of small effect size, whose frequencies vary among populations, use of these measures to detect emerging resistance may not have the power to tease apart natural heritable variation in pesticide tolerance (Pujol et al., 2018) from variation caused by exposure to other environmental factors (Fitt et al., 1998; Hoy et al., 1998; McKenzie, 2000). Changes in crop damage caused by a pest may also be used to identify resistant populations, but none of these approaches document resistance heritability, which is critical for classifying a population as resistant (Tabashnik et al., 2013).

### 5.2 F1 and F2 progeny testing

F1 and F2 progeny testing were developed to address the challenge of demonstrating heritability and quantifying changes in rare resistance‐causing mutations. They require establishing crosses between field‐collected pests and measuring resistance phenotypes of their offspring in laboratory bioassays. While this approach can document heritability, quantifying differences in pest phenotypes requires months of work for rearing and testing field‐collected pests and their offspring (Tabashnik et al., 2006). Moreover, there is a significant tradeoff between effort spent on the number of field‐collected parents and the number of offspring that can be tested. Given the finite time and resources available for bioassays, fewer field‐collected samples can be tested as more offspring are used for resistance detection.

### 5.3 Molecular monitoring of monogenic resistance

To move beyond these limitations, research has focused on understanding the genetic basis of resistance so that adaptive changes in pest DNA could be tracked. In most cases, resistance mutations have been discovered from laboratory‐selected populations or from screening wild resistant populations. Field populations are then examined for these same resistance mutations using polymerase chain reaction‐based methods (Gahan et al., 2007; Jin et al., 2018; Morin et al., 2003; Zhang et al., 2013). Yet quantifying changes in the frequency of target‐site mutations has limitations: tracking changes in resistance based on a single gene or mutation can only identify resistance if most pest populations or species have converged on the same resistance mechanisms. Disparities between resistance‐conferring mutations in lab‐selected and field‐collected populations of *Drosophila melanogaster* are well documented and underscore this problem (reviewed in Ffrench‐Constant, 2013). In the case of Bt resistance, multiple genes (Jurat‐Fuentes et al., 2021; Liu et al., 2010), and even multiple species‐specific mutations within a gene, have the potential to confer resistance (Fabrick et al., 2014; Morin et al., 2003; Xie et al., 2005). Detection of resistance‐conferring mutations in one pest population may allow mutation‐specific molecular monitoring in other pest populations targeted by the same management practices but is only useful when resistance mechanisms are shared. Where this is not the case, failure to identify changes at known gene targets of selection may mislead researchers and regulatory agencies as resistance emerges by novel genetic mechanisms in pest populations.

### 5.4 Monitoring polygenic pesticide resistance

Early resistance monitoring advocates proposed that quantitative genetic approaches could be used to monitor changes in pest resistance arising from polygenic adaptation (Firko & Hayes, 1990; Via, 1986). Using techniques such as offspring‐parent regression and sibling analysis, researchers proposed estimating additive genetic variance (*V*_A_) and narrow‐sense heritability (*h* ^2^), related through the following equation:

$$h^{2}=V_{A}/V_{P},$$

where *V*_P_ is the total phenotypic variation in a population. As selection occurs over generations, *V*_A_ and *h* ^2^ are expected to decline as resistance mutations replace existing genetic variation from susceptible individuals. Monitoring populations for this decline was thought to be one approach to tracking changes in polygenic resistance (Firko & Hayes, 1990). However, wide confidence intervals surrounding *V*_A_ and *h* ^2^ estimates (Firko & Hayes, 1990) and an inability to account for sources of environmental and nonadditive genetic variation (Pujol et al., 2018) may interfere with the use of quantitative genetic models for resistance monitoring. Furthermore, *V*_A_ and *h* ^2^ estimates are specific to the environments in which they are measured so that the choice of dose, type of observation (e.g., mortality, egg production, larval growth), and the timing of measurements following the deployment of a novel pesticide would all impact estimates of *V*_A_ and *h* ^2^ and how they change over time (Firko & Hayes, 1990). For these reasons, adopting quantitative genetic approaches for polygenic resistance monitoring is rare.

## 6 GENOMIC TOOLS HAVE THE POTENTIAL TO PLAY A CRITICAL ROLE IN RESISTANCE MONITORING

Altogether, these challenges highlight a critical need for a monitoring approach to measure changes in the resistance status of a pest caused by one or many mutations. Recent advances in sequencing technology may fulfill this critical monitoring need. Genome scanning approaches enabled by high throughput sequencing (Box 3) have the potential to detect early adaptive allele frequency changes that establish pest populations on an evolutionary trajectory toward economically important resistance. To detect these changes, allele frequencies of present‐day samples could be compared to samples collected at the time of deployment for a new pesticide technology. Moreover, allele frequency changes in field‐collected individuals could be coupled to resistance measurements made at the time of collection. Such an approach could link changes in the frequencies of known and unknown resistance mutations to growing pesticide resistance phenotypes in wild pest populations.

BOX 3 **Strategies for SNP marker generation**

With unlimited resources, whole‐genome resequencing (WGRS) is optimal for SNP generation in genome scanning studies. It allows for investigating changes in genetic variants across the entire genome. However, for routine resistance monitoring, cost‐effectiveness may be important when choosing an approach. Two additional library preparation and sequencing approaches for SNP generation include reduced representation library (RRL) sequencing and pooled sequencing (Pool‐seq). Advantages and limitations of each SNP generation approach must be considered when examining genomic responses to selection (Fuentes‐Pardo & Ruzzante, 2017; Lowry et al., 2017; McKinney et al., 2017; Tiffin & Ross‐Ibarra, 2014).

**RRL approach**

This library preparation and sequencing strategy only generates SNPs from a fraction of an organism's genome (<15%). Common RRL preparation strategies include genotyping‐by‐sequencing (GBS; Elshire et al., 2011) and restriction‐site associated DNA‐sequencing (RAD‐seq; Davey & Blaxter, 2010). Here, I include exome‐based sequencing in my definition of RRL libraries because sequencing only the exome reduces the representation of the genome sequenced per individual in an experiment. Generating SNP markers from a fraction of an organism's genome makes it cost‐effective to collect genotypic information from many individuals. However, the number of genotyped individuals is traded for the density of markers throughout the genome, limiting the percentage of the genome for which responses to selection can be quantified (Tiffin & Ross‐Ibarra, 2014).

**Pool‐seq approach**

For this library preparation and sequencing strategy, many individuals with similar phenotypes, from similar environments or from the same field collection timepoints, can be pooled and sequenced together to cover each locus in the genome to a depth of <1× coverage per individual (Schlötterer et al., 2014). SNP frequencies can then be calculated for pools rather than at the individual level, exchanging the ability to collect genotype information from each individual sequenced for lower sequencing costs. The accuracy of estimated allele frequencies can be poor if pool sizes are low and unreplicated (Anand et al., 2016; Schlötterer et al., 2014).

## 7 DETECTING RAPID GENOMIC RESPONSES TO NATURAL SELECTION

Over the past 20 years, the number of studies aimed at detecting rapid genome‐wide responses to selection in nonmodel organisms has grown (Ahrens et al., 2018; Hansen et al., 2012). Genome scanning approaches are uncovering the basis for local adaptation without a priori knowledge of the specific trait under selection or its genetic architecture (Tiffin & Ross‐Ibarra, 2014). Once genomic targets of selection are identified, knowledge of gene function can be used to infer the phenotype under selection. Revolutionary advances in the methods and tools of evolutionary biology have enabled this proliferation of studies and include (1) the development of novel marker generation approaches for nonmodel organisms (Davey & Blaxter, 2010; Fuentes‐Pardo & Ruzzante, 2017; Schlötterer et al., 2014), (2) reduced costs of next‐generation sequencing, and (3) an expansion of genome assemblies in nonmodel metazoan lineages (Ellegren, 2014; Fuentes‐Pardo & Ruzzante, 2017). Nucleic acids are isolated from individuals exposed to different selection pressures and prepared in high‐throughput sequencing libraries (Box 3). Once sequencing reads are generated and aligned to a reference genome, genomic targets of selection are identified based on differences in the frequency and diversity of individual SNPs or groups of SNPs inherited together (i.e., haplotypes) for samples collected in the presence of different selective pressures.

### 7.1 Models of genome‐wide responses to selection

Models of genomic response to selection fall within two extremes: (1) the hard selective sweep model, for which strong selection at a single gene could be considered the most extreme case, and (2) the polygenic model of adaptation (Barghi et al., 2020). Under the hard selective sweep model, a locus under selection experiences an increase in the frequency of an adaptive allele and a loss of neutral or deleterious alleles. A “selective sweep” of neutral DNA sequences that flank (i.e., are physically near) the locus under selection accompanies the selected allele, resulting in a “footprint” of selection much broader than the target mutation alone (i.e., haplotype; Nielsen, 2005). Selective sweeps can be “hard,” resulting in a single haplotype at a target of selection (Nielsen, 2005), or “soft,” where a few haplotypes linked to an adaptive allele increase in frequency (Messer & Petrov, 2013). Both produce footprints of selection that are identifiable as greater than expected allele frequency change at a locus relative to average change observed in selected and unselected populations across the rest of the genome (Beaumont & Nichols, 1996; Tiffin & Ross‐Ibarra, 2014) and/or longer than expected stretches of low genetic diversity in a selected population (Pavlidis & Alachiotis, 2017).

Under the alternative polygenic model, phenotypic changes in a population are driven by tiny shifts in allele frequency at hundreds or thousands of loci (Fisher, 1930). While it has been suggested that most adaptive traits have a polygenic basis (Rockman, 2012), most studies that have detected true polygenic signatures of selection using genome scanning approaches have examined artificially selected populations in otherwise controlled environments (e.g., Barghi et al., 2019; Castro et al., 2019; Kelly & Hughes, 2019; Therkildsen et al., 2019; Turner et al., 2011). Empirical studies of natural selection documenting genome‐wide polygenic signatures of selection according to Fisher (1930) exist (Bergland et al., 2014; Chaturvedi et al., 2021), but are rare (Connallon & Hodgins, 2021). Between these extremes is an oligogenic model of adaptation, where a few mutations of moderate to large effect size explain most of a trait's variation (Barghi et al., 2020), but one to many mutations of small effect may modify trait expression as a population moves toward its adaptive optimum (Connallon & Hodgins, 2021). It seems likely that selection in natural systems leads to a range of adaptive patterns at the genomic level.

### 7.2 Potential and pitfalls of genome scanning under different models of molecular evolution

Genome scanning studies are correlative in nature (Tiffin & Ross‐Ibarra, 2014) and can document allele frequency changes in response to selection at one or many loci. To date, most genome scanning studies identify adaptive allele frequency shifts by comparing populations exposed to different selection pressures in space (e.g., latitudinal clines). Yet tracking changes within the same population before and after exposure to a novel selection pressure provides the most powerful evidence of response to selection. In natural systems, temporal genome scanning experiments have identified strong allele frequency shifts over the course of a few to dozens of generations (e.g., Bergland et al., 2014; Bi et al., 2013, 2019; Campbell‐Staton et al., 2017; Chaturvedi et al., 2021; Ergon et al., 2019; Mikheyev et al., 2015; Schiebelhut et al., 2018; Stahlke et al., 2021). Further study (e.g., QTL analysis, genome‐wide association studies [GWAS]) is required to causally link putative targets of selection to adaptive phenotypes (Jones et al., 2012; Price et al., 2018; Tiffin & Ross‐Ibarra, 2014). A small but growing number of studies have taken this extra step to establish or link published functional support to candidate targets of selection (Bergland et al., 2014; Campbell‐Staton et al., 2017; Chaturvedi et al., 2021; Mikheyev et al., 2015; Stahlke et al., 2021), documenting that strong adaptive temporal shifts in allele frequency at multiple genomic loci can be observed on short time scales. Although sampling intervals varied by study (range = ca. 1–30 generations), these studies documented allele frequency shifts that were detectable above background genomic change in tens of generations. This suggests that genome scanning could be applied to measure allele frequency shifts corresponding to pesticide use on the short time scales required for genomic resistance monitoring.

Detecting the weak adaptive shifts in allele frequency that accompany true polygenic adaptation remains a challenge related to statistical power, however (Barghi et al., 2020; Kemper et al., 2014). Large numbers of samples collected prior to and post selection are required to document small allele frequency changes over background genomic variation. Furthermore, quantitative genomic approaches used for functional confirmation have poor resolution to detect allele‐specific impacts on a trait when its architecture is truly polygenic and also require very large sample sizes (Crouch & Bodmer, 2020). Temporal genome scanning studies that have successfully detected polygenic adaptation have focused on organisms that are easily collected and for which generating large sample sizes is feasible: wild *Drosophila melanogaster* (Bergland et al., 2014) and *Daphnia magna* (Chaturvedi et al., 2021). Documenting polygenic adaptive responses using genome scanning may continue to be a challenge for difficult‐to‐sample or low abundance species, including some pests. Although pests are problematic due to their abundance in agroecosystems, the feasibility of collecting large numbers of pests following deployment of a novel pesticidal technology, but prior to resistance evolution, may depend on the pest species, its host range, mode of pesticide delivery (plant incorporation vs. sprayable), rates of adoption and application, etc. Understanding where field‐evolved pesticide resistance falls within the continuum of models of genome‐wide response to selection will help determine the role that genome scanning can play in the future of resistance monitoring.

## 8 DOES SELECTION BY PESTICIDES DIFFER FROM SELECTION IN NATURAL SYSTEMS?

It has been suggested that the human‐imposed selection pressures found in agroecosystems, including pesticides, are stronger than (Connallon & Hodgins, 2021; Palumbi, 2001) and distinct from selection pressures experienced by organisms in natural systems (Thrall et al., 2010). Therefore, organismal responses to selection in agro‐systems may also differ from those observed in natural systems (Thrall et al., 2010). Moreover, loss of biodiversity and widely adopted population management practices homogenize agricultural landscapes and increase habitat connectivity over that of natural systems. This may impact population demographic responses, such as population expansions and contractions, or facilitate long‐distance movement of pests and resistant genotypes (Dauer et al., 2009). Important questions are whether major differences in responses to selection exist between agroecosystems and natural systems, and whether any such differences would impact detection of genome‐wide responses to selection by pesticides at known resistance‐conferring loci.

Selection coefficients range from 0 to 1 and quantify the extent to which selection is acting to reduce the contribution of a wild‐type allele relative to an adaptive allele to the gamete pool in subsequent generations. Previous studies of the strength of response to selection by pesticides, including in agroecosystems, estimate that selection coefficients (s) range from 0.16 to 1 using both phenotypic and single locus genotypic data from field‐evolved populations (Groeters & Tabashnik, 2000; Milesi et al., 2016). In natural systems, a meta‐analysis by Thurman and Barrett (2016) examined 3416 estimates of selection coefficients (s) from 79 studies. Mean and median s values across all studies were 0.135 and 0.082, respectively, and there were 112 estimates of s that exceeded 0.5 (max = 1.0). Many of the highest s values were associated with transplant experiments of the wild mustard, *Boechera stricta* (Anderson et al., 2014), and the insect, *Timema cristinae* (Gompert et al., 2014), which examined shifts in trait means and allele frequencies that occurred in transplanted populations over time.

Fritz et al. (2018) calculated s values for SNPs across the genome of *Chloridea* (*Heliothis*) *virescens*, a major agricultural pest of cotton managed by synthetic pesticides, which evolved strong resistance to pyrethroids in the 1990s. When pyrethroid pressure was reduced following commercialization of Bt crops in 1996, Fritz et al. (2018) found that the frequency of the resistance‐conferring L1029H mutation declined, likely due to its fitness cost in the absence of pyrethroids. This decline was detectable above between‐year variation across the genomic background of field‐collected *C. virescens*. Moreover, the value of *s* calculated for selection against L1029H was 0.03 in a single generation for populations of *C. virescens* from Louisiana and Texas. At other loci that underwent greater than expected temporal change, per generation selection coefficients ranged from 0.007 to 0.737. The magnitude of s depended on starting allele frequency, degree of dominance, and as in Thurman and Barrett (2016), the time period over which s was calculated (Fritz et al., 2018). Key takeaways from comparisons of these studies are that organisms in natural and agroecosystems show significant variation in responses to selection across the genome, but values of s estimated for pesticide resistance loci, and those from across the genome of *C. virescens* did not strongly differ from those reported in natural systems (Thurman & Barrett, 2016).

## 9 GENOME SCANNING TO DETECT PESTICIDE RESISTANCE IN AGROECOSYSTEMS

Recent successful detection of genomic responses to selection in natural systems over the course of a few generations (Bergland et al., 2014; Campbell‐Staton et al., 2017; Chaturvedi et al., 2021; Mikheyev et al., 2015; Stahlke et al., 2021) as well as evidence that the strength and detectability of responses to selection do not necessarily differ between natural and agricultural systems suggests that genomic approaches hold promise for detection of resistance evolution. Indeed, several recent studies have used genome scanning of field‐collected pests to identify genes under selection by pesticides (Calla et al., 2021; Crossley et al., 2017; Gimenez et al., 2020; Kamdem et al., 2017; Pélissié et al., 2022; Taylor et al., 2021; Weedall et al., 2020). Yet few have examined the same arthropod populations in an agroecosystem over time to reveal adaptive genomic change in response to pest management practices.

One such study by Taylor et al. (2021) examined entire genomes of wild *Helicoverpa zea*, a major agricultural insect pest. Wild North American *H. zea* have evolved damaging levels of resistance to the Cry toxins expressed in Bt crops. Using WGS and RRL data from hundreds of *H. zea*, Taylor et al. (2021) identified regions of the *H. zea* genome that underwent significant allele frequency changes over a 15‐year period (2002–2017) following commercialization of Bt‐expressing crops. Some of these changes, which arose concurrently with field‐evolved phenotypic resistance, overlapped with resistance QTL identified from F2 mapping families. Moreover, QTL analysis revealed both the genomic architecture of field‐evolved Cry resistance and the effect sizes of genomic regions involved. Allele frequency changes with major impacts on Cry1Ab resistance were identified on three chromosomes, and the genomic basis of Cry2Ab2 resistance was isolated to one chromosome (Taylor et al., 2021). Loci of small effect size also contributed to Cry resistance, altogether indicating an oligogenic architecture of resistance. Although several candidate genes for Bt resistance have been identified in other lepidopteran pests, no strong allele frequency shifts occurred in these candidate genes in temporal field collections of *H. zea*. This finding of temporal changes at many novel resistance‐associated genomic regions in an agricultural pest demonstrates the importance of moving beyond candidate genes to genome‐wide resistance monitoring.

Genome scanning also revealed that the number and magnitude of adaptive genomic changes in *H. zea* increased between 2012 and 2016, years in which damage to Bt‐expressing crops also accelerated (Dively et al., 2016). Yet major allele frequency changes in regions of the genome linked to Cry resistance were detectable as early as 2012, prior to documentation of widespread field failures of Bt crops caused by *H. zea*. To date, no specific genes have been identified as necessary and sufficient for *H. zea* resistance to Bt crops. Identifying the specific genes involved in resistance may not be necessary for monitoring, however. Documenting temporal allele frequency changes in a genomic region and confirming linkage of that region with an insecticide resistance phenotype should be sufficient to trigger regulatory action. Although identifying the genetic mechanism of resistance may be of academic interest, this could be pursued in follow‐up studies.

## 10 POTENTIAL AND PITFALLS OF GENOMIC MONITORING FOR RESISTANCE EVOLUTION IN AGROECOSYSTEMS

Development of pest management tools requires years of effort and significant financial investment. Resistance monitoring programs have the potential to offer significant benefits to the agricultural community if they can efficiently identify and mitigate the evolution of widespread resistance (Gould, 1978). Genomic tools have strong potential to detect allele frequency changes that accompany resistance evolution (Beckie et al., 2021; Gould et al., 2018). Taylor et al. (2021) demonstrated that genome scanning can detect emerging resistance using samples collected from the same sites over a 15‐year period following deployment of a novel pest management tool. Long‐term collections of pests and proper archival of specimens to preserve DNA were critical tools for their study. Results from Taylor et al. (2021) also documented the importance of monitoring in a gene‐agnostic way by demonstrating that regions of strong temporal allele frequency change did not include candidate genes for Bt resistance. Yet several potential challenges to widespread use of this approach were also identified. Not all major allele frequency shifts were associated with responses to selection by the Bt toxins used in their study. This highlights the importance of linking genomic change to a resistance phenotype of interest when applying genome scanning to detect emerging resistance, particularly for pests managed with multiple pesticides. Simple linkage analyses targeting SNPs in regions of major allele frequency change or genome‐wide approaches like QTL analysis or GWAS could be used for functional confirmation. Another strategy could involve building the functional analysis into the monitoring study design. For example, replicated temporal collections from paired treated and untreated plots could be used for monitoring with the expectations that (1) allele frequencies in resistance‐conferring genomic regions should differ by treatment and (2) resistance‐conferring regions should show increased allele frequency divergence over time. However, temporal allele frequency changes may appear more stochastic among replicates if they result from genetic drift.

Another major challenge to genomic monitoring would be identifying resistance‐associated allele frequency shifts in pests with strong population genomic structuring. Such genome‐wide population structure leads to inaccurate detection of allele frequency changes, particularly for analytical approaches that rely upon empirically derived genome‐wide thresholds for statistical significance (Pérez‐Figueroa et al., 2010). Accommodating strong population genomic structure in genome scans is challenging, although analytical tools that attempt to minimize the influence of this structure are available (e.g., Ma et al., 2015). Fortunately, *H. zea* is a migratory species, with high levels of standing genetic variation and little population genomic structure (Seymour et al., 2016), which made it feasible to detect resistance‐associated allele frequency shifts against background genome‐wide change (Taylor et al., 2021).

Many other agricultural insect pests share these qualities with *H. zea*: they have high levels of standing genetic variation as measured by rates of polymorphism (Fritz et al., 2016; Schoville et al., 2018) and effective population sizes (Anderson et al., 2018; Daly & Gregg, 1985) and experience high levels of gene flow (Groot et al., 2011; Kim et al., 2011; Miller et al., 2009). Yet some pests exhibit strong population genetic structure (Schoville et al., 2018). When sampled geographically, such structure could hamper detection of resistance evolution by using temporal genome scanning. Careful sampling design, including use of paired treated and untreated plots in more geographic locations, as well as analytical approaches to accommodate population genetic structure (Stahlke et al., 2021) may be important if genomic monitoring is to be used for species with low rates of migration and gene flow (see below).

### 10.1 What is needed for further development of genomic monitoring for pesticide resistance?

Detecting temporal allele frequency shifts by genome scanning requires mapping reads to well‐assembled and annotated pest genomes. As of 2020, there were >500 insect genomes assembled and publicly available, including many major pest species (Guo et al., 2022). However, many of these assemblies are fragmented, which could hamper detection of hard selective sweeps for pesticide resistance using linkage disequilibrium‐based approaches. Recently, the USDA announced the Ag100 pest initiative, with the goal of producing high‐quality genome assemblies for the top 100 agricultural pests in the U.S. (Childers et al., 2021). This resource‐generating effort will facilitate future population and evolutionary genomic studies of pests, as well as application of genomic approaches for resistance monitoring.

A second issue is that population genomic structure of pest species could hamper detection of significant allele frequency shifts, particularly when using analytical approaches that rely upon empirically derived genome‐wide thresholds for statistical significance. If there is major divergence across the genomes of pests collected over time, it could lead to increased genome‐wide significance thresholds and ultimately inaccurate detection of temporally diverging allele frequencies. For pest species that exhibit major spatial population genomic structure (e.g., Schoville et al., 2018), it will be important to understand whether they show similar patterns of strong genome‐wide divergence over years. If such divergence exists over years, it may be difficult to use temporal genome scanning to accurately detect emerging resistance through subtle changes in allele frequencies. Likewise, strong spatial population genomic structure might suggest that resistance alleles would spread slowly through the landscape, resulting in islands of highly resistant populations. Under these circumstances, the feasibility of temporal genomic monitoring would rely upon the geographical extent of sampling and the probability that resistance alleles can be found in sampled regions. Therefore, for those spatially structured pests, understanding the extent of genome‐wide divergence over space and time, as well as estimating migration rates between populations, will be crucial to determining whether genomic monitoring is feasible.

Finally, detection of contemporaneous responses to selection associated with emerging pesticide resistance is much more important than understanding how genomes have responded to past episodes of selection. Many recently published genome scanning analyses focus on detecting genomic changes after phenotypic shifts have already been observed. The goal of genomic monitoring for resistance, however, is to identify resistance before it is widespread, and with sufficient time to take remedial action to preserve pesticidal technologies. Additional research is needed to better understand the frequency with which field sampling and sequencing should occur to pre‐emptively detect pesticide resistance evolution.

## CONFLICT OF INTEREST

The author has no conflict of interest to declare.

## Data Availability

Data sharing is not applicable to this article as no new data were created or analyzed in this study.
